# CFTR heterozygosity in severe asthma with recurrent airway infections: a retrospective review

**DOI:** 10.1186/s13223-022-00684-0

**Published:** 2022-06-06

**Authors:** Eldar Priel, Adil Adatia, Melanie Kjarsgaard, Parameswaran Nair

**Affiliations:** 1 McMaster University Department of Medicine, Hamilton, Canada; 2 Firestone Institute for Respiratory Health, St Joseph’s Healthcare Hamilton, 50 Charlton Avenue East, Hamilton, ON L8N 4A6 Canada

**Keywords:** CFTR, Cystic fibrosis, Severe asthma, Sputum neutrophils

## Abstract

**Rationale:**

Patients with asthma who have neutrophilic bronchitis may have an underlying cause leading to increased susceptibility to airway infections.

**Methods:**

Retrospective review of patients with asthma who had a previous history of recurrent exacerbations that had been associated with airway or sinus infections referred to a tertiary asthma center between 2005 and 2020. Demographics, clinical features, and airway inflammation type determined by sputum cytometry were compared between CFTR carriers and non-carriers. Multiple linear regression was used to identify clinical predictors of CFTR carrier status. Response to nebulized hypertonic saline was assessed by comparing the number of infective exacerbations before and after its initiation.

**Results:**

75 patients underwent *CFTR* mutation testing. Of these, 13 (17%) were CFTR carriers. The most common mutation was $\Delta$F508. CFTR carriers were older (adjusted odds ratio 1.06 (CI 95% 1.01, 1.13)) and had more frequent flares requiring hospitalization (4.19 (1.34, 24.74)). Neutrophilic airway inflammation was the most common inflammatory subtype in CFTR carriers, though 8/13 also had eosinophilic bronchitis. Nebulized hypertonic saline was well tolerated by most and reduced the frequency of infective exacerbations.

**Conclusions:**

The prevalence of *CFTR* heterozygosity in this cohort with recurrent neutrophilic bronchitis is higher than in the general population. Respiratory disease in CFTR carriers is associated with older age and may cause significant morbidity. Airway neutrophilia is the most common inflammatory subtype, but > 50% had eosinophilic bronchitis requiring treatment. Hypertonic saline appears to be well tolerated and effective in reducing the number of infective exacerbations.

**Supplementary Information:**

The online version contains supplementary material available at 10.1186/s13223-022-00684-0.

## Introduction

Asthma is characterized by variable airflow limitation and is usually associated with airway inflammation. The luminal cellular inflammatory component can be characterized based on the proportion of eosinophils and neutrophils found in the sputum [1]. Asthmatics with neutrophilic or mixed eosinophilic-neutrophilic bronchitis tend to have more severe disease with worse lung function, higher glucocorticoid requirements, and greater health care utilization compared to asthmatics with eosinophilic inflammation alone [2]. Airway infections can be the primary driver of sputum neutrophilia with increased total cellularity and poor asthma control in these patients, and they should be investigated for conditions leading to increased susceptibility to infection [3, 4].

One potential cause of increased susceptibility to airway infections in asthma patients is cystic fibrosis transmembrane conductance regulator (CFTR) hypofunction due to heterozygosity for a damaging *CFTR* mutation. Individuals who are heterozygous for a disease-causing mutation in *CFTR* (CF carriers), typically have normal sweat chloride, and have been considered protected from the manifestations of cystic fibrosis [5]. However, a number of authors have reported that CF carriers are overrepresented in cohorts of certain respiratory diseases such as diffuse bronchiectasis [6, 7], chronic rhinosinusitis [8], and asthma [9].

We previously reported the clinical histories of three asthma patients who were CF carriers and had persistent neutrophilic bronchitis [10]. In the present study, we sought to examine the prevalence of CF carriers in patients with asthma referred with a history of infective exacerbations and to characterize the clinical characteristics of CF carriers, to identify clinical predictors of CF carrier status in patients with severe airways disease, and assess the efficacy and safety of hypertonic saline in these patients. Our hypothesis was that persistent neutrophilic bronchitis, bronchiectasis and certain bacterial infections will be predictors for CFTR mutation carriage.

## Methods

### Patient data

We conducted a retrospective study of patients with a physician diagnosis of asthma who were referred for further evaluation to the Firestone Institute of Respiratory Health (FIRH) airway disease clinic in Hamilton, Ontario between August, 2005 and July, 2020 for recurrent infectious exacerbations (two or more in the previous 12 months). As a part of our diagnostic workup of susceptibility to infections, we examined CFTR mutation analysis in these regardless of previous diagnosis of cystic fibrosis or family history. Ethics approval was obtained from the Hamilton Integrated Research Ethics Board. All charts of patients who underwent *CFTR* genetic testing were reviewed. Spirometry, quantitative sputum cell counts, sputum culture, methacholine provocation concentration causing a 20% reduction in forced expiratory volume in 1 s (PC20), chest computed tomography (CT) findings (ascertained from a radiologist report), and serum immunoglobulin concentration data were collected. Genetic analysis was performed by The Hospital for Sick Children molecular diagnostics laboratory, which uses multiplex polymerase chain reaction (PCR) to test for the 39 most common pathogenic *CFTR* variants in Caucasian populations (Additional file 1: Table S1) and analyzes the intron 8 polythymidine genotype (9 T, 7 T, or 5 T), using direct mutation assays [11]. In Certain patients, CFTR Gene sequencing was carried by the lab using direct sequence analysis of the coding and flanking region of CFTR gene.

Concurrent asthma was determined based on compatible symptoms and at least one confirmatory test (bronchodilator response > 200 mL and > 12% in forced expiratory volume in 1 s (FEV1), PC20 ≤ 8 mg/mL, or ≥ 15% reduction in FEV1 with nebulized saline during sputum induction). Chronic rhinosinusitis was identified based on sinonasal symptoms and confirmation either by nasal endoscopy performed by an otolaryngologist or by CT sinuses.

### Sputum analysis

Sputum cytometry was performed for every patient at least once, as is the protocol of our clinic. Sputum was induced by inhalation of hypertonic saline, and the expectorate was processed to obtain the total cell count and cell differential using standard methodology [12]. Eosinophilic bronchitis was defined as > 3% sputum eosinophils, neutrophilic bronchitis was defined as total cell count of at least 15 × 10^6^ cells/g with at least 65% neutrophils, and pleiotropic bronchitis was defined as the presence of both eosinophilic and neutrophilic bronchitis. A patient was considered to have persistent neutrophilia if they had > 2 episodes of neutrophilic bronchitis and was considered to have persistent intense neutrophilia if they had > 2 episodes of neutrophilic bronchitis in which the neutrophil differential exceeded 85%. An exacerbation was defined as a change in symptoms and lung function from a subject’s usual status [13]. In light of previous studies [4, 14], an infectious exacerbation was defined as an exacerbation where a bacterial culprit was identified in cultures, or when sputum cytometry showed neutrophilia and the patient responded to antibiotics.

### Response to hypertonic saline

In our clinic, hypertonic saline (3–7%, nebulized, twice daily) is used to attenuate and prevent recurrent infectious exacerbations. The use of nebulized hypertonic saline facilitates airway clearance, reduction of bacterial burden and mitigates stasis. The change in number of infective exacerbations between the year prior to and after starting hypertonic saline in patients was documented and reviewed from the medical chart.

### Statistical analysis

Patient demographics, clinical features, and laboratory data were summarized using descriptive statistics. Differences in baseline characteristics between CF carriers and non-carriers were compared using two-sided t-tests for continuous variables and Fisher’s Exact Test for categorical variables. The number of neutrophilic exacerbations before and after hypertonic saline was compared using the Wilcoxon signed-rank test. A significance level of < 0.05 was used for all inferences. The association between different patient characteristics and CF carrier status was assessed using multivariable logistic regression. Backwards stepwise regression was performed using the Akaike information criterion to select the optimal regressors. Analyses were performed using R (R Core Team, 2021) and GraphPad Prism 9.0 (GraphPad Software, Inc., San Diego, CA).

## Results

### Patient characteristics

There were 75 patients referred with physician-diagnosed asthma who had *CFTR* mutation testing. This represents approximately 50% of all patients with asthma and at least one neutrophilic exacerbation, and 20% of all patients in our clinic. Table 1 shows the clinical characteristics of each group. *CFTR* mutations were identified in 13/75 patients (17.3%). This is significantly greater than expected assuming a *CFTR* mutation prevalence of 4% in the general population (p = 0.01). CF carriers were older (67 years (SD 13.2) compared to 56 years (14.7), p < 0.01) on average compared to non-carriers. FEV1% predicted was similar between the two groups (65.7% in carriers and 69.0% in non-carriers, p = 0.65). There was a numerical trend to increased prevalence of bronchiectasis (70.0% vs 41.9%) and chronic rhinosinusitis (85.7% vs 33.8%) in CF carriers, but this did not reach statistical significance. Alternate causes of increased susceptibility to airway infections including lymphopenia (frequently associated with oral corticosteroid use) and hypogammaglobulinemia were comparable between both groups (p = 0.551 for lymphopenia and p = 1.00 for need for intravenous immunoglobulin therapy).

**Table 1:** Clinical and laboratory characteristics of the CF carrier and non-carrier groups

	CF carriers N = 13	Non-carriers N = 62	p value
	Mean (SD)/%	Mean (SD)/%	
Age (years)	67 (13.2)	56 (14.7)	< 0.01
Male (%)	38	23	1.00
BMI > 25 kg/m^2^ (%)	54.5	65.9	1.00
FEV1 (L)	1.76 (0.80)	2.13 (0.84)	0.17
FEV1 (% predicted)	65.7 (22.7)	69.0 (24.7)	0.65
Ever smokers (%)	50.0	57.0	1.00
Atopy (%)	50.0	67.7	0.09
Chronic rhinosinusitis (%)	85.7	33.8	0.52
Bronchiectasis (%)	70.0	41.9	0.54
Asthma (%)	46.2	50.0	1.00
Hospitalizations/year	0.54 (1.23)	0.11 (0.34)	0.19
% With persistent neutrophilia	58.3	44.4	0.52
% With persistent intense neutrophilia	58.3	35.2	0.19
Max neutrophil count (cells × 10^6^/g)	56.5 (61.0)	40.1 (45.0)	0.37
Max sputum eosinophils (%)	8.0 (10.3)	7.9 (15.11)	0.57
Eosinophilic bronchitis (%)	58.0	40.7	0.34
Lymphopenia (%)	61.50	50.9	0.55
IVIG (%)	25	22.6	1.00
Daily OCS (%)	30.7	35.6	1.00
HTS (%)	76.9	30.0	0.004
Azithromycin (%)	23.1	11.1	0.36

Persistent neutrophilia was defined as at least two episodes of neutrophilic bronchitis. Persistent intense neutrophilia was defined as at least 2 episodes with a sputum total cell count > 15 × 10^6^ cells/g and a neutrophil differential > 85%

### Sputum cytometry

Neutrophilic bronchitis was the most common sputum finding in both groups. There was a non-significant trend towards increased intensity of neutrophilic bronchitis in CF carriers (56.5 vs 40.1 cells × 10^6^/g, p = 0.37) and higher prevalence of persistent intense neutrophilia (58.3% vs 35.2%, p = 0.19). A similar proportion of both groups also had eosinophilic bronchitis (58.0% in CF carriers and 40.7% in non-carriers, p = 0.34), and approximately one-third in each group required daily oral corticosteroid therapy for optimal control.

### CFTR mutations

The clinical characteristics of each CF carrier are shown in Table 2. The most prevalent mutation in our cohort was phenylalanine-508 deletion ($\Delta$F508), which was found in 7 patients. The other mutations, each appearing once in our cohort, were c.1157T>A, R117H, 621 + 1G>T, c.2249C>T, c.1540G>A, and S549N. Three CF carriers had an intron 8 polypyrimidine 5 T *CFTR* allele, including 1 patient who was $\Delta$F508 heterozygous. This patient had the lowest FEV1 and highest burden of infections, including pneumonia due to multidrug resistant *Pseudomonas*. Of those with no detectable *CFTR* mutation, seven carried the intron 8 tract 5 T allele. No differences in the prevalence of neutrophilic exacerbations or persistent neutrophilia were detected between those with or without an intron 5 T allele in the absence of a *CFTR* mutation (p = 0.43, p = 0.69, respectively).

**Table 2:** Clinical characteristics and treatment of CF carriers with recurrent airway infections

#	Age (yrs)	Sex (M/F)	BMI (kg/m^2^)	CFTR mutation	Intron 8 alleles	FEV1 (L) (% pred)	Asthma^a^ (y/n)	Bronchiectasis (y/n)	Max sputum eos (%)	Sputum culture	Change in inf/yr with HTS
1	51	F	35.8	$\Delta$F508	7 T/9 T	N/A^b^	N/A	N	6.5	SA/ST	− 1
2	75	M	25.0	$\Delta$F508	9 T/9 T	2.96 (94)	Y	Y	0	–	− 4
3	51	F	32.9	$\Delta$F508	7 T/9 T	2.4 (81)	N	N	34.5	–	+ 2
4	78	M	25.8	$\Delta$F508	7 T/9 T	1.82 (65)	N	N	5	HiB/NC/PA	− 4
5	47	M	33.8	$\Delta$F508	7 T/9 T	1.28 (50)	Y	N	5.8	HiB/NTM	+ 0
6	69	F	26.2	$\Delta$F508	5 T/9 T	0.32 (16)	N	Y	0.8	NTM/PA	N/A^c^
7	70	F	24.9	$\Delta$F508	7 T/9 T	2.28 (88)	Y	Y	37.8	HiB/MC/NTM/PA/SP	− 3
8	75	F	26.0	c.1175 T>A	7 T/7 T	1.03 (61)	N	N	2	–	− 1
9	77	F	23.2	R117H	5 T/7 T	1.54 (75)	Y	Y	19	–	+ 0
10	54	F	40.0	621 + 1G > T	7 T/9 T	1.17 (48)	Y	N	14.3	–	N/A^c^
11	62	M	31.6	c.2249C>T	7 T/9 T	1.60 (47)	N	Y	1.5	–	− 3
12	76	M	21.9	c.1540G>A	5 T/7 T	3.15 (90)	Y	Y	6.3	–	+ 0
13	90	F	23.1	S549N	7 T/9 T	1.52 (73)	N	Y	0	–	N/A

HiB, *Haemophilus influenzae* type b; HTS, hypertonic saline; NC, Nocardia; NTM, non-tubercular mycobacterium; PA, *Pseudomonas aeruginosa*; SA, *Staphylococcus aureus*; SP, *Streptococcus pneumoniae*; ST, Stenotrophomonas

^a^Represents presence of current asthma at the time of evaluation based on bronchodilator reversibility (FEV1 > 12% and > 200 mL improvement with bronchodilator administration) or methacholine PC20 < 8 mg/mL

^b^Patient had tracheostomy placed due to hereditary angioedema with recurrent laryngeal attacks, so spirometry was not obtained

^c^Patient 6 was not treated with hypertonic saline due to low FEV1 and Patient 10 did not tolerate it due to bronchoconstriction

### Sputum microbiology

Microbiological data is presented in Table 3. The most prevalent isolated organism was Pseudomonas aeruginosa in 17 patients (23%), followed by Haemophilus influenzae (17.6%). there was no difference between CFTR mutation carriers and non carriers.

**Table 3:** Prevalent bacterial organisms isolated from sputum samples

Organism	Number (%)
*Pseudomonas aeruginosa*	17 (23%)
*Hemophilus influenza* B	13 (17.6%)
Non-*Tuberculous mycobacteria*	9 (12.2%)
Aspergillus* species	7 (9.5%)
*Streptococcus pneumoniae*	7 (9.5%)
*Staphylococcus aureus*	7 (9.5%)
*Moraxella catarrhalis*	6 (8.1%)
*Klebsiella pneumoniae*	1 (1.3%)
*Stenotrophomonas maltophilia*	1 (1.3%)

*No subject had an invasive fungal infection

### Response to hypertonic saline

**Fig. 1:**
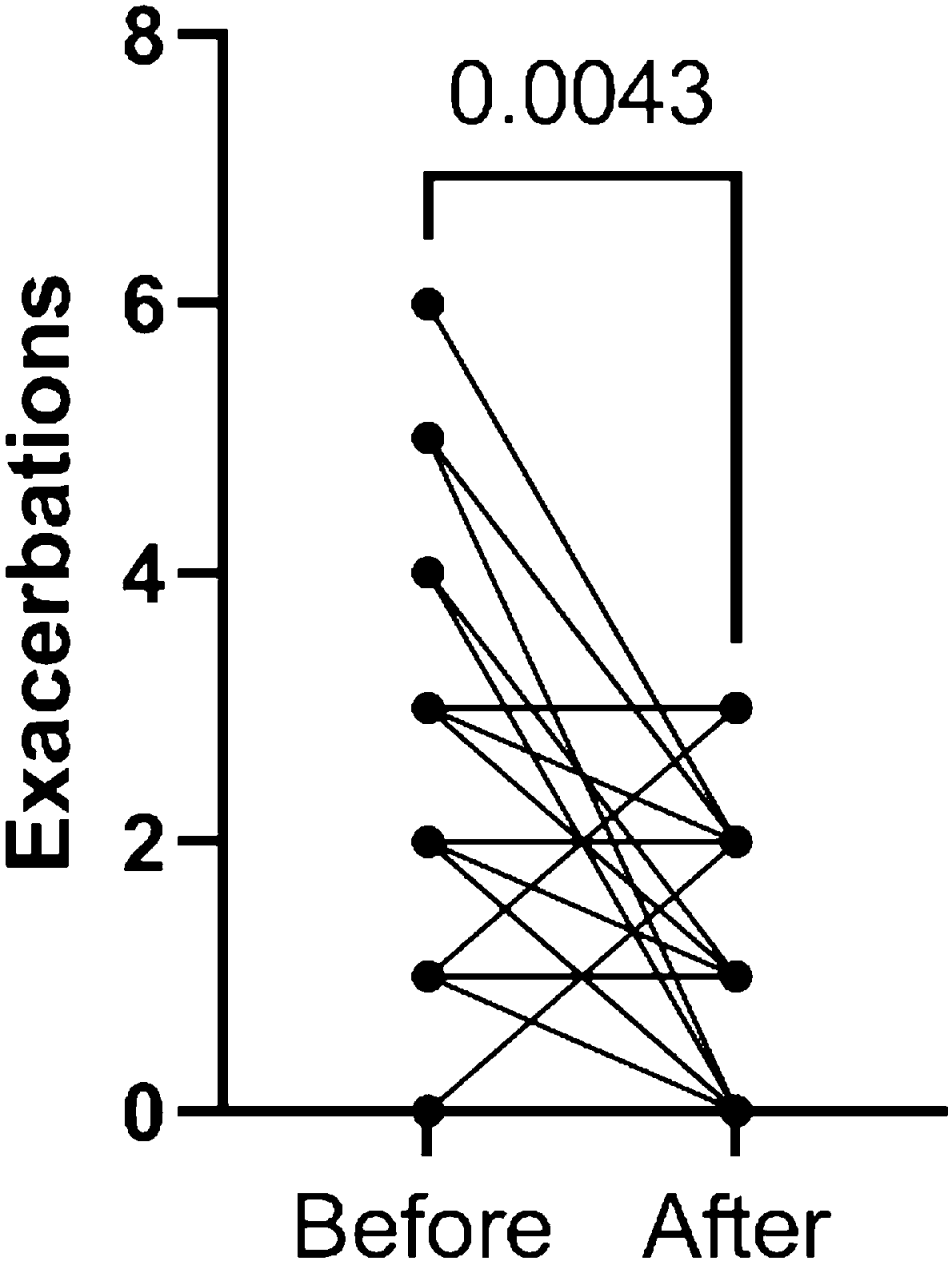
Number of infective exacerbations in the year before and after hypertonic saline initiation in patients with physician-diagnosed asthma (n = 29). Each dot represents the number of yearly exacerbations before and after HTS. Each line represents the trend. Several subjects have similar numbers of exacerbations, therefore 29 patients generated 16 trends

The change in number of infective exacerbations between the year prior to and after starting hypertonic saline in patients with physician-diagnosed asthma is shown in Fig. 1. Data were available for 29/75 individuals (19 non-carriers and 10 CF carriers). The median number of infective exacerbations was 2.0 in the year prior to hypertonic saline and was significantly reduced in the year after to a median of 1.0 (p = 0.0043). One additional patient was not prescribed hypertonic saline due to poor FEV1 and another did not tolerate it due to 5% saline induced bronchoconstriction (which caused an 18% reduction in FEV1). The average reduction in FEV1 with nebulized saline was 4.9% (S.D. 7.4%). Anecdotally, the patients with concomitant sinus disease with or without bronchiectasis who have the delta508 mutation seem to derive the maximum benefit.

### Predictors for CF carrier status

Two independent variables were found to be significant predictors of CFTR heterozygosity—age and number of exacerbations needing hospitalization per year (Table 4). The adjusted odds ratio for age was 1.06 (95% C.I. 1.01, 1.13) for each additional year of age. The adjusted odds ratio for hospitalization was 4.19 (1.34, 24.74) per hospitalization. A history of polymicrobial infection (at least two different pathogens identified by sputum culture on two different occasions), bronchiectasis, chronic rhinosinusitis, and number neutrophilic or pleiotropic flares were not significant predictors. Backwards stepwise regression identified age and hospitalizations per year as the most important parameters.

**Table 4:** Multivariate comparison between CF carrier and non-carrier groups

Parameter	Adjusted odds ratio (95% CI)	p value
Age	1.06 (1.01, 1.13)	0.03^†^
Polymicrobial infection	0.47 (0.07, 2.36)	0.39
Chronic rhinosinusitis	1.68 (0.40, 7.24)	0.47
Bronchiectasis	0.99 (0.23, 4.22)	0.99
Number of neutrophilic flares	1.14 (0.87, 1.45)	0.28
Number of pleiotropic flares	0.86 (0.05, 5.56)	0.88
Hospitalizations per year	4.19 (1.34, 24.74)	0.04^†^

Polymicrobial infection was defined as at least two different pathogens identified by sputum culture on two different occasions. A neutrophilic flare was diagnosed when an increase in symptoms was accompanied by sputum total cell count > 15 × 10^6^ cells/g and neutrophils > 65% on cell differential. A pleiotropic flare was similarly diagnosed when sputum total cell count > 15 × 10^6^ cells/g, neutrophils > 65%, and eosinophils > 3% on cell differential

^†^Indicates a significant difference between CF carriers and non-carriers at p < 0.05

## Discussion

We conducted a retrospective chart review to characterize clinical features of CF carriers with asthma and recurrent neutrophilic inflammation. Our main findings are: (i) CF carriers are overrepresented in this cohort, supporting the role of CFTR hypofunction in the predisposition of some patients with asthma to recurrent respiratory infections, (ii) the dominant inflammatory type in these patients is neutrophilic bronchitis, but some have eosinophilic inflammation as well, and (iii) hypertonic saline appears to be effective and is well tolerated in this asthmatic population.

We identified pathogenic *CFTR* mutations in 17% of subjects. The carrier rate of pathogenic *CFTR* gene mutations is approximately 4% in persons of northern European descent [15], indicating a more than fourfold higher prevalence of CF carriers in our cohort, which may be an underestimation since not all of the included patients were from high risk ethnic groups. This higher prevalence should be seen in light of previous findings in this field, challenging the dogma that CF carriers are asymptomatic. CF carriers have also been found to have a higher prevalence of chronic rhinosinusitis compared to the general population [16]. A large Danish population study that assessed patients specifically for the $\Delta$F508 variant found that CF carriers were at increased risk of chronic bronchitis and bronchiectasis [17]. Additionally, a large American database study found that CF carriers were at increased risk of recurrent airway infections and many other manifestations of CF [18]. Though these reports suggest that the absolute risk is low, 3–4% of the population are CF carriers in the USA, Canada, and Northern Europe [15], and the potential morbidity caused by *CFTR* heterozygosity in these populations is large. Most recently, the CFTR gene was sequenced in participants from the severe asthma research program (SARP), and potentially pathogenic CFTR variants were found in 9.5% of this severe asthma-enriched cohort [20]. Our findings add further evidence that CF carriers are at increased risk of recurrent pulmonary infections and show that these patients may have significant morbidity and health care utilization with frequent hospitalizations.

Three of the 13 CF carriers had the intron 8 splice variant 5 T. This variant is located at a splice acceptor site of intron 8 and causes frequent skipping of exon 9, leading to a dysfunctional CFTR protein. Compound heterozygotes with $\Delta$F508 and 5 T have significantly reduced CTFR function and a high rate of symptoms attributable to CF, including pulmonary disease and congenital bilateral absence of vas deference [20]. In our cohort, patient 6 was compound heterozygous for these variants and it is possible that her much more severe respiratory disease compared to the other $\Delta$F508 carriers was due to the 5 T variant. Further data are needed to determine the importance of the intron 8 polypyrimidine genotype in symptomatic adult CF carriers.

Consistent with our previous study [10], neutrophilic bronchitis was the dominant sputum inflammatory subtype in CF carriers, which reflects recurrent or persistent airway infection in these patients. However, 8/13 CF carriers also had eosinophilic bronchitis, and the prevalence of eosinophilic bronchitis was similar between CF carriers and non-carriers. These patients required inhaled corticosteroids, and in some cases systemic corticosteroids and anti-eosinophil biologicals for optimal management. Therefore, even though CF carriers are at greater risk of airway infections, flares of respiratory symptoms in these patients should not be assumed to be due to infection. Sputum cell counts are needed to determine whether anti-inflammatory or antibiotic therapy is needed.

In this cohort, asthmatic CF carriers with recurrent infections were older on average compared to non-carriers at the time of diagnosis. The genetic and environmental factors that cause a small subset of CF carriers to develop pulmonary disease have yet to be fully elucidated, and it is possible these factors accumulate over time to produce disease manifestations in certain patients. Evidence supporting this hypothesis includes studies showing that CFTR hypofunction can be acquired from cigarette smoke exposure [21], bacterial and viral infections [22, 23], and neutrophilic inflammation [24]. It is thus possible that a positive feedback loop ensues with worsening CFTR function caused by infection and neutrophil recruitment to the airways, leading to further infections. In addition to recurrent infections, CFTR hypofunction may also contribute to asthma by increasing airway smooth muscle contractility by modulating cellular calcium mobilization [25]. A longitudinal study examining respiratory symptoms and CFTR function in CF carriers is needed to test this hypothesis. However, it is important to consider *CFTR* mutations even in older patients presenting later in life with exacerbations of asthma associated with recurrent airway infections.

In our cohort, nebulized hypertonic saline was well tolerated in asthma patients despite reduced FEV1 and demonstrable bronchial hyperresponsiveness in some patients. This is an important finding, as there are little data on the safety of hypertonic saline in asthma and these data are limited to mild-to-moderate asthma [26, 27]. There was also a significant reduction in the number of infective exacerbations, and some experienced up to five fewer infections in the year after initiation. Presently, pharmacologic therapy for uncontrolled, severe asthma with neutrophilia is limited to macrolide therapy [3]. It thus seems reasonable to trial nebulized hypertonic saline in asthmatics with recurrent respiratory infections, potentially in addition to macrolide therapy, in the absence of more definitive evidence supporting treatment choices. Given that hypertonic saline is effective in reducing the frequency of airway infections in CF [28], CF carriers with asthma may benefit more than asthmatic non-carriers, but this requires further study. Recently, CFTR modulators have been approved for $\Delta$F508 homozygous patients and such therapies may benefit CF carriers as well, though the cost is prohibitive [29]. Novel therapeutic strategies such as inhibition of the epithelial Na^+^ channel in the airway may also prove to be helpful in CF carriers in the future [30].

This study has several important limitations. First, this is a single center retrospective chart review from a tertiary airway disease clinic, so the morbidity in CF carriers observed is influenced by selection bias and not generalizable to the full CF carrier population. The cohort described represents 20% of our volume, therefore selecting patients for CFTR genotyping could have influenced the results. Second, *CFTR* mutations were detected using a multiplex PCR panel rather than gene sequencing, so rare *CFTR* variants may have been missed. This could have led to misclassification of CF carriers as non-carriers or true CF patients with compound heterozygosity as CF carriers. However the genetic screen used would detect mutations in over 84% of a pan-ethnic north American population [31], so this is unlikely to have affected the results. Third, for most of our cohort, a sweat chloride test was not available, so it is possible some CF carriers could have met the diagnostic criteria for CF or CF related disorder. Fourth, some CF carriers had other additional factors that contributed to airway infections such as hypogammaglobulinemia needing immunoglobulin substitution therapy and oral corticosteroid use, though these factors were not more common in the CF carriers compared to non-carriers. Fifth, though all patients in this cohort were referred with a physician-diagnosis of asthma, current asthma could not be confirmed in all patients. Most patients at the time of referral had a FEV1 below 65% predicted, so a methacholine challenge could not be performed. Sixth, since *CFTR* mutation analysis was obtained only in patients with recurrent neutrophilic bronchitis, this analysis is limited to those patients with patients referred for asthma who have infective exacerbations. If a broader asthma population were considered, the significance of factors such as bronchiectasis and non-tuberculous mycobacterial disease in predicting CF carrier status may have been more apparent. Finally, given the retrospective nature of the study and the small number of CF carriers studied, our conclusions regarding the safety and efficacy of nebulized hypertonic saline should be considered provisional.

In summary, CF carriers are significantly overrepresented in a cohort of asthma patients with recurrent airway infections referred to a tertiary care centre, and we recommend testing for CFTR mutations in patients with asthma with recurrent airway infections, even in older patients. Neutrophilic bronchitis is the dominant inflammatory type, but some also have concomitant eosinophilic bronchitis that may be severe enough to warrant treatment with an anti-IL5 biological. Hypertonic saline was well tolerated in most patients. Additional research is needed to assess the efficacy of hypertonic saline and other treatment strategies in asthmatics with exacerbations associated with intense neutrophilic bronchitis.

## Supplementary Information

**Additional file 1: Table S1.** The 39 pathogenic variants tested for using multiplex PCR.

## Data Availability

The datasets used during and/or analysed during the current study will be made available from the corresponding author on reasonable request.

## Abbreviations

CF: Cystic fibrosis
CFTR: Cystic fibrosis transmembrane conductance regulator
FEV1: Forced expiratory volume in 1 s
PC20: Methacholine provocation concentration causing a 20% reduction in FEV1
PCR: Polymerase chain reaction
