# Supplementary material for: CFTR heterozygosity in severe asthma with recurrent airway infections: a retrospective review
Source: Allergy Asthma Clin Immunol. 2022 Jun 6;18:46. doi: 10.1186/s13223-022-00684-0 (PMC9172019; doi:10.1186/s13223-022-00684-0)
Supplement: Supplementary file 1 — Additional file 1: Table S1. The 39 pathogenic variants tested for using multiplex PCR. [file 13223_2022_684_MOESM1_ESM.docx]

Table S1- The 39 pathogenic variants tested for using multiplex PCR

| G85E^*^ | G542X^*^ | R117H^*^ | W1282X^*^ | 3659delC^*^ | 3876delA | DeltaF508^*^ | 3120+1G>A |
| --- | --- | --- | --- | --- | --- | --- | --- |
| S549N^*^ | R334W^*^ | A455E^*^ | R1162X^*^ | 711+1G>T^*^ | 1078delT^*^ | DeltaI507^*^ | 1898+5G>T |
| R347P^*^ | Y122X | R347H | S1255X | 394delITT | 2307insA | 1898+1G>A^*^ | S549R(T>G) |
| R553X^*^ | R560T^*^ | A559T | Y1092X | 2184delA^*^ | 2183AA>G | 2789+5G>A^*^ | 3849+10kbC>T^*^ |
| V520F | G551D^*^ | N1303K^*^ | M1101K | 3905insT | 621+1G>T^*^ | 1717-1G>A^*^ |  |

^*24 common cystic fibrosis mutations included in the minimum mutation panel prior to 2002 (11)^
